# Supplementary material for: Complementary networks of cortical somatostatin interneurons enforce layer specific control
Source: eLife. 2019 Mar 18;8:e43696. doi: 10.7554/eLife.43696 (PMC6422636; doi:10.7554/eLife.43696)
Supplement: Transparent reporting form [file elife-43696-transrepform.docx]

***eLife’s* transparent reporting form**

We encourage authors to provide detailed information *within their submission* to facilitate the interpretation and replication of experiments. Authors can upload supporting documentation to indicate the use of appropriate reporting guidelines for health-related research (see [EQUATOR Network](http://www.equator-network.org/%20)), life science research (see the [BioSharing Information Resource](https://biosharing.org/)), or the [ARRIVE guidelines](http://www.plosbiology.org/article/info:doi/10.1371/journal.pbio.1000412) for reporting work involving animal research. Where applicable, authors should refer to any relevant reporting standards documents in this form.

If you have any questions, please consult our Journal Policies and/or contact us: [editorial@elifesciences.org](mailto:editorial@elifesciences.org).

**Sample-size estimation**

- You should state whether an appropriate sample size was computed when the study was being designed
- You should state the statistical method of sample size computation and any required assumptions
- If no explicit power analysis was used, you should describe how you decided what sample (replicate) size (number) to use

Please outline where this information can be found within the submission (e.g., sections or figure legends), or explain why this information doesn’t apply to your submission:

A Monte Carlo simulation was used to calculate the number of paired recordings to be performed for the experiments in figure 4 as well as the optimal sampling strategy (whether to record many independent pairs versus to test multiple connections onto a single cell).

For optogenetic slice experiments and optogenetic in vivo experiments, we did not explicitly perform power analyses in advance, but estimated the number of animals that would be required based on our previous work (Pluta et al 2015) which used similar methods. This required an assumption that the effect size of our manipulations would be at least as large as in that work.

**Replicates**

- You should report how often each experiment was performed
- You should include a definition of biological versus technical replication
- The data obtained should be provided and sufficient information should be provided to indicate the number of independent biological and/or technical replicates
- If you encountered any outliers, you should describe how these were handled
- Criteria for exclusion/inclusion of data should be clearly stated
- High-throughput sequence data should be uploaded before submission, with a private link for reviewers provided (these are available from both GEO and ArrayExpress)

Please outline where this information can be found within the submission (e.g., sections or figure legends), or explain why this information doesn’t apply to your submission:

Description of replicates can be found in the methods section as well as in the corresponding figure legends for each experiment.

For experiments in which we recorded multiple trials of physiology data from the same neurons (either in slice or in vivo, Fig. 1 – 6), trials could be considered technical replicates whereas different neurons could be considered biological replicates. In general, datapoints and descriptions of N in the main text/figure legends refer to biological replicates. For analyses where we recorded technical replicates in the form of multiple trials, we performed analyses either by

1) averaging over trials (technical replicates) for each neuron (biological replicate) so that each neuron counted for n = 1 in our statistical sample 2) explicitly accounting for the hierarchical structure (e.g. multiple neurons in each slice/mouse recorded) of our data using mixed-effects models or 3) performing statistical tests via resampling-based permutation tests. For single-cell RNA sequencing, we performed sequencing on samples generated by pooling tissues from multiple mice in the same batch – thus multiple mice were part of a biological replicate for these experiments.

Exclusion criteria for physiology and transcriptomic experiments can be found in the corresponding portions of the methods section. Besides these exclusion criteria, we did not exclude any outliers from our analyses post-hoc.

Our sequencing data and analysis code can be found at https://github.com/drisso/x94.

**Statistical reporting**

- Statistical analysis methods should be described and justified
- Raw data should be presented in figures whenever informative to do so (typically when N per group is less than 10)
- For each experiment, you should identify the statistical tests used, exact values of N, definitions of center, methods of multiple test correction, and dispersion and precision measures (e.g., mean, median, SD, SEM, confidence intervals; and, for the major substantive results, a measure of effect size (e.g., Pearson's r, Cohen's d)
- Report exact p-values wherever possible alongside the summary statistics and 95% confidence intervals. These should be reported for all key questions and not only when the p-value is less than 0.05.

Please outline where this information can be found within the submission (e.g., sections or figure legends), or explain why this information doesn’t apply to your submission:

We report p-values, N, statistical tests used, and basic descriptive statistics in the main text, as well as in supplementary table 1. Definition of error bars can be found in the figure legends. Details for more complicated analyses can be found in the methods.

(For large datasets, or papers with a very large number of statistical tests, you may upload a single table file with tests, Ns, etc., with reference to sections in the manuscript.)

**Group allocation**

- Indicate how samples were allocated into experimental groups (in the case of clinical studies, please specify allocation to treatment method); if randomization was used, please also state if restricted randomization was applied
- Indicate if masking was used during group allocation, data collection and/or data analysis

Please outline where this information can be found within the submission (e.g., sections or figure legends), or explain why this information doesn’t apply to your submission:

Our samples were allocated into experimental groups on the basis of their genotype. In most cases this did not allow us to randomize or blind the experimenters to the identity of the animal being used, since typically tissue of these animals was fluorescent and it was not possible to avoid observing this while performing experiments. For in vivo cre-dog experiments, we injected an entire litter with AAV cocktails and used non-GFP expressing littermates as control animals.

Masking was not used during data analysis, but in cases where we needed to apply exclusion criteria to data (e.g. when excluding mice that did not run enough during in vivo experiments, or when excluding cells with unacceptable changes in access resistance during slice experiments) we performed quality control steps prior to analyzing any data.

**Additional data files (“source data”)**

- We encourage you to upload relevant additional data files, such as numerical data that are represented as a graph in a figure, or as a summary table
- Where provided, these should be in the most useful format, and they can be uploaded as “Source data” files linked to a main figure or table
- Include model definition files including the full list of parameters used
- Include code used for data analysis (e.g., R, MatLab)
- Avoid stating that data files are “available upon request”

Please indicate the figures or tables for which source data files have been provided:

Source data files are available for Figure 7 as well as supplementary figure 14
